# Klotho Inhibits Unilateral Ureteral Obstruction-Induced Endothelial-to-Mesenchymal Transition *via* TGF-β1/Smad2/Snail1 Signaling in Mice

**DOI:** 10.3389/fphar.2019.00348

**Published:** 2019-04-05

**Authors:** Shasha Li, Lixia Yu, Aolin He, Qifeng Liu

**Affiliations:** ^1^ Clinical Research and Lab Centre, Kunshan First People’s Hospital Affiliated to Jiangsu University, Kunshan, China; ^2^ Department of Nephrology, Kunshan First People’s Hospital Affiliated to Jiangsu University, Kunshan, China

**Keywords:** Klotho, unilateral ureteral obstruction, endothelial-mesenchymal transition, transforming growth factor-β1, Snail1

## Abstract

This study aimed to evaluate repression of Klotho on unilateral ureteral obstruction (UUO)-induced renal fibrosis and endothelial-to-mesenchymal transition (EndoMT) in mice. Renal fibrosis model was established by UUO in C57BL/6 male mice. Recombinant Klotho protein was administered to UUO mice as treatment group, and the mice in sham and UUO group were administered with an equal volume of vehicle. EndoMT biomarkers and TGF-β1/Smad2/Snail1 signaling were examined by immunofluorescence, immunohistochemistry, and western blotting assays. UUO deteriorated kidney function and resulted in increased expression of the mesenchymal marker α-smooth muscle actin and decreased expression of vascular endothelial cadherin, an endothelial marker. Moreover, UUO enhanced TGF-β1, phosphorylated Smad2 (p-Smad2), and Snail1 expression. Interestingly, Klotho treatment suppressed UUO-induced TGF-β1, p-Smad3, and Snail1 expression, which was accompanied by alleviation of the EndoMT process. Our findings demonstrated that Klotho significantly ameliorated EndoMT progression by targeting TGF-β1/Smad/Snail1 signaling in UUO mice, which provides the possibility for Klotho-based therapeutic protection against renal fibrosis.

## Introduction

The *klotho* gene, which was discovered as an aging-suppressor gene, encodes two Klotho proteins including membranous (mKlotho) and soluble (sKlotho) forms (Kuro-o et al., 1997; van Loon et al., 2015). Klotho protein declines in diseased kidney tissue of patients with chronic kidney disease (CKD) (Shimamura et al., 2012; Liu et al., 2018) and animal models with renal interstitial fibrosis (Kim et al., 2015; Hu et al., 2016; Kuro, 2017). Our group (Liu et al., 2018) and others (Doi et al., 2011; Shimamura et al., 2012) have confirmed that loss of Klotho protein aggravated kidney fibrosis, while Klotho overexpression improved kidney function and ameliorated renal fibrosis. Intriguingly, the supplement of exogenous Klotho could alleviate renal fibrosis by inhibiting endoplasmic reticulum stress and EMT (Liu et al., 2015, 2017), targeting canonical TGF-β1 (Doi et al., 2011) or Wnt/β-catenin signaling (Satoh et al., 2012), or the renin-angiotensin system (Zhou et al., 2015). Thus, Klotho has been identified as a novel fibrotic target for intervention in renal fibrotic disorders (Doi and Masaki, 2017).

Although the etiologies of renal fibrosis are vastly different, it shares the common feature of persistent activation and accumulation of myofibroblasts. Generally, activated myofibroblasts can originate from resident fibroblasts, bone marrow fibrocytes, or the transition of other cells into myofibroblasts either by epithelial-to-mesenchymal transition (EMT) or endothelial-to-mesenchymal transition (EndoMT) (Sun et al., 2016). Increasing evidence suggest a close correlation of EndoMT with renal fibrosis, as it plays a significant role in producing myofibroblast cells (He et al., 2013; Cruz-Solbes and Youker, 2017). During the course of pathology, endothelial cells that undergo EndoMT lose their specific markers, such as vascular endothelial cadherin (VE-cad) or platelet endothelial cell adhesion molecule (PECAM-1/CD31), and gain myofibroblast markers, such as α-smooth muscle actin (α-SMA) (Piera-Velazquez et al., 2016). Inhibition of EndoMT can reverse the endothelial phenotype and retard renal fibrosis (Deng et al., 2016); thus, targeting EndoMT has emerged as a novel therapeutic strategy for renal fibrosis (Li et al., 2010; Cruz-Solbes and Youker, 2017). Transforming growth factor (TGF)-β1 is known to be the strongest driver for organ fibrosis, and both TGF-β1 protein and its downstream signaling are believed to exert a crucial role in inducing EndoMT (Pardali et al., 2017).

In our previous study, we have demonstrated that the amelioration of renal fibrosis was associated with the suppression of renal EMT in the cyclosporine A-treated rats (Liu et al., 2017). Similar to EMT, EndoMT is also a complex process associated with the emergence of myofibroblasts and renal fibrosis. However, it remains unclear whether Klotho can be ascribed to amelioration of EndoMT. Hence, our study examines whether Klotho inhibits EndoMT *via* TGF-β1 signaling in UUO mice.

## Materials and Methods

### UUO Model and Klotho Administration

Thirty-eight-week-old C57BL/6 male mice were supplied from Shanghai Sipper-BK Laboratory Animal Co. (Shanghai, China). All procedures were approved by the Animal Care and Use Committee of our hospital. All animals were housed with a 12-h light/dark cycle and free access to water and standard commercial rat chow. Mice were randomly designated into three groups (*n* = 6 per group): sham, model, and Klotho treatment. Renal fibrosis was induced by UUO, as described in our previous study (Liu et al., 2015). All experimental mice underwent the UUO procedure except for mice in the sham group (whereby the ureter was not obstructed). Recombinant mouse Klotho protein (R&D systems) was administered to mice in the Klotho treatment group by intraperitoneal injection at a dosage of 0.02 mg/kg every other day starting immediately after UUO operation (Liu et al., 2015). Mice in sham and model groups were injected with physiological saline. Body weight was monitored and the dosage of agents was calculated based on body weight. On day 14 after UUO operation, blood samples were collected and obstructed kidneys were rapidly removed from mice (under anesthetization) and washed with saline. Obstructed kidneys were processed for histopathological examination, immunofluorescence, immunohistochemistry, and western blotting analysis.

### Biochemical Parameter Analysis

Blood samples were drawn on day 14 for determination of serum creatinine (Scr) and blood urea nitrogen (Bun) with an automatic biochemical analyzer (Roche Modular P, Basel, Switzerland).

### Histopathological Analysis

Hematoxylin/eosin (HE) and Masson’s trichrome staining methods were performed to assess pathological changes of renal tissues. Obstructed kidneys were fixed in formalin, dehydrated in ethanol, and embedded in paraffin. Next, 3-μm-thick sections were produced from processed tissues and stained with HE and Masson’s trichrome. Stained slices were evaluated for pathological changes by microscopy (Olympus BX51, Tokyo, Japan) at 400× magnification. Image-Pro Plus v6.0 software (Media Cybernetics, USA) was used to evaluate the degree of renal fibrosis by two investigators in a blinded manner. For assessment of tubulointerstitial fibrosis, the proportion of blue-stained fibrotic areas was evaluated.

### Immunofluorescent Staining

Renal tissue sections (4-μm-thick) were fixed in acetone at −20°C for 10 min and blocked with 5% bovine serum albumin after washing with phosphate-buffered saline (PBS) for 1 h. Next, renal tissue sections were incubated with rabbit anti-VE-cad monoclonal antibody (mAb) (1:200, Abcam, Cambridge, UK), and mouse anti-α-SMA mAb (1:200, Abcam) overnight at 4°C. After washing with PBS, tissues were incubated with secondary antibodies (fluorescein isothiocyanate-labeled goat anti-rabbit antibody and Cy3-labled goat anti-mouse antibody; Beyotime, Nantong, China) for 1 h at room temperature, and counterstained with 4′,6-diamidino-2-phenylindole (DAPI, Beyotime, Nantong, China) for 10 min to visualize nuclei. The primary antibody was replaced with PBS as a negative control. Images were viewed and analyzed at 400× magnification using immunofluorescence microscopy (Olympus BX51) and Image-Pro Plus 6.0. Ten visual fields per kidney were analyzed for co-localization of VE-cad and α-SMA.

### Immunohistochemical Staining

Paraffin-embedded kidney tissue slices were incubated with primary rabbit antibodies against TGF-β1 and phosphorylated (p)-Smad2 (S255) (all from Abcam), followed by incubation with horseradish peroxidase (HRP)-conjugated secondary antibody (Beyotime). Images were obtained using an Olympus BX51 microscope at 400× magnification.

### Western Blotting Analysis

Protein samples were extracted from kidney tissues and their concentrations were quantified using a BCA kit (Thermo Scientific, Waltham, MA). The protein was isolated and loaded into an SDS-PAGE gel, and then transferred to PVDF membranes, which were incubated with primary antibodies against Snail1, TGF-β1, and phosphorylated Smad2 (p-Smad2, S255) overnight at 4°C after blocking with 5% milk in TBST. Next, membranes were incubated with HRP-conjugated secondary antibody for 2 h at room temperature. GAPDH or β-tubulin was used as an internal control for normalization of relative protein levels. Antigen-specific signals were examined and quantified using VisionWorks software (Analytik, Jena, Germany).

### Statistical Analysis

Continuous variables are presented as mean ± standard deviation and were analyzed with one-way ANOVA. Differences between groups were compared by the least significant difference test. A *p* ≤ 0.05 was considered statistically significant. Statistical analysis was carried out using SPSS 23.0 software (IBM, Armonk, NY).

## Results

### Klotho Administration Attenuated UUO-Induced Klotho Reduction in Mice

Klotho was expressed mainly by the kidney, and significantly decreased in UUO mice, compared with the sham group (Figures 2B,C). However, Klotho treatment significantly increased renal Klotho expression (Figures 2B,C). As Klotho, a kidney-protective factor, is reduced, Klotho replacement therapy may have beneficial effects for kidney diseases.

### Klotho Administration Improved Kidney Function and Attenuated Renal Fibrosis in UUO Mice

**Figure 1:**
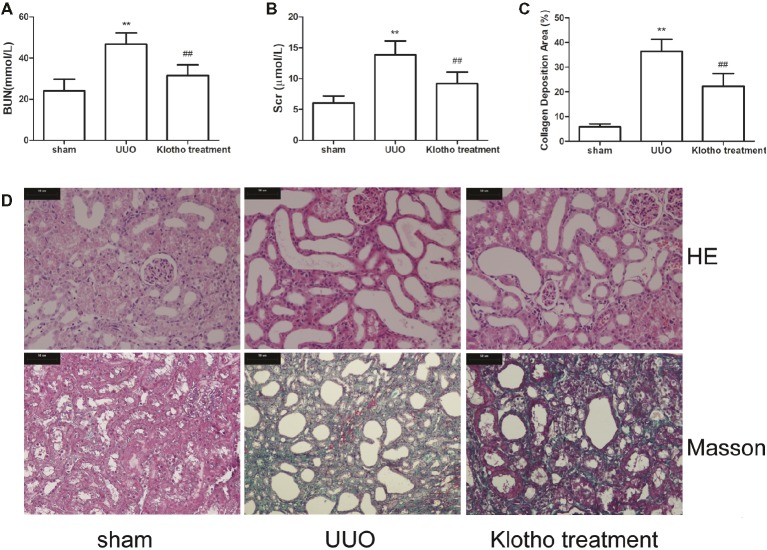
Renal function and tubulointerstitial fibrosis were reduced by Klotho administration in UUO mice. **(A)** Blood urea nitrogen alteration in experimental groups. **(B)** Serum creatinine alteration in experimental groups. **(C)** Quantification of tubulointerstitial fibrosis. **(D)** Representative images of renal fibrosis were analyzed by HE and Masson’s trichrome staining in experimental groups. **p ≤ 0.01 versus sham group; ^##^ *p* ≤ 0.01 versus UUO group.

UUO mice had the highest levels of Bun and Scr (Figures 1A,B). However, levels of Bun and Scr were notably decreased after Klotho treatment (Figures 1A,B).

Based on HE staining images, it was shown that kidneys in UUO mice showed tubular dilatation, edema, inflammatory cell infiltration, and interstitial fibrosis (Figure 1D). However, these pathological changes were considerably ameliorated by Klotho treatment (Figure 1D).

Based on Masson’s staining images, kidneys in sham group mice showed almost no areas with interstitial fibrosis. In contrast, kidneys in all UUO mice exhibited large areas of interstitial fibrosis (Figures 1C,D). However, the degree of interstitial fibrosis was remarkably reduced by Klotho treatment. These data indicated that Klotho effectively protected kidney function in UUO mice (Figures 1C,D).

### Klotho Restricted the EndoMT Process in Fibrotic Kidney

**Figure 2:**
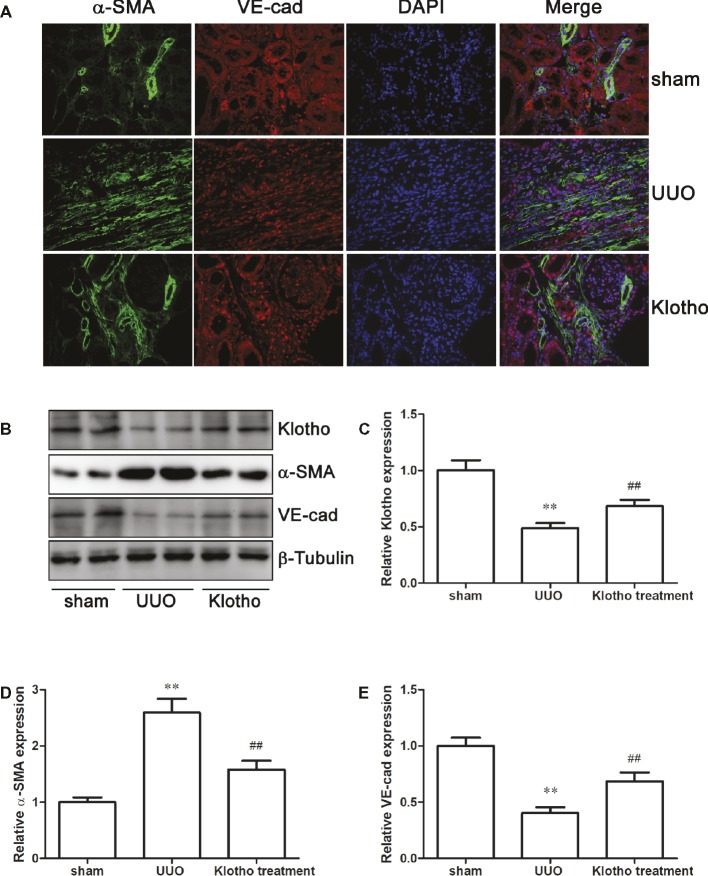
Klotho administration reversed EndoMT process in UUO mice. **(A)** Immunofluorescence staining demonstrates α-SMA (green), VE-cad (red), and DAPI (blue) in experimental groups. **(B)** Representative western blotting image of Klotho, α-SMA, and VE-cad expression in experimental groups. β-Tubulin expression was analyzed as an endogenous control. Densitometric analysis was performed for quantification of the bands of Klotho **(C)**, α-SMA **(D),** and VE-cad **(E)**. **p ≤ 0.01 versus sham group; ^##^ *p* ≤ 0.01 versus UUO group.

The immunofluorescence data revealed a decreased number of VE-cad-expressing cells and increased number of α-SMA-expressing cells in UUO kidney compared with sham-operated mice (Figure 2A). Additionally, we observed that VE-cad-expressing cells acquired α-SMA staining in UUO kidneys (Figure 2A). Consistently, western blotting analysis showed a loss of VE-cad and increase of α-SMA in fibrotic kidneys (Figures 2D,E). These changes indicated the occurrence of EndoMT in fibrotic kidney. Interestingly, disrupted expression of VE-cad and α-SMA was partially restored by Klotho treatment (Figures 2A,D,E). Taken together, these data suggest that Klotho may inhibit the EndoMT process in obstructed kidneys.

### Klotho Inhibited the TGF-β1/Smad2/Snail 1 Signaling Pathway

**Figure 3:**
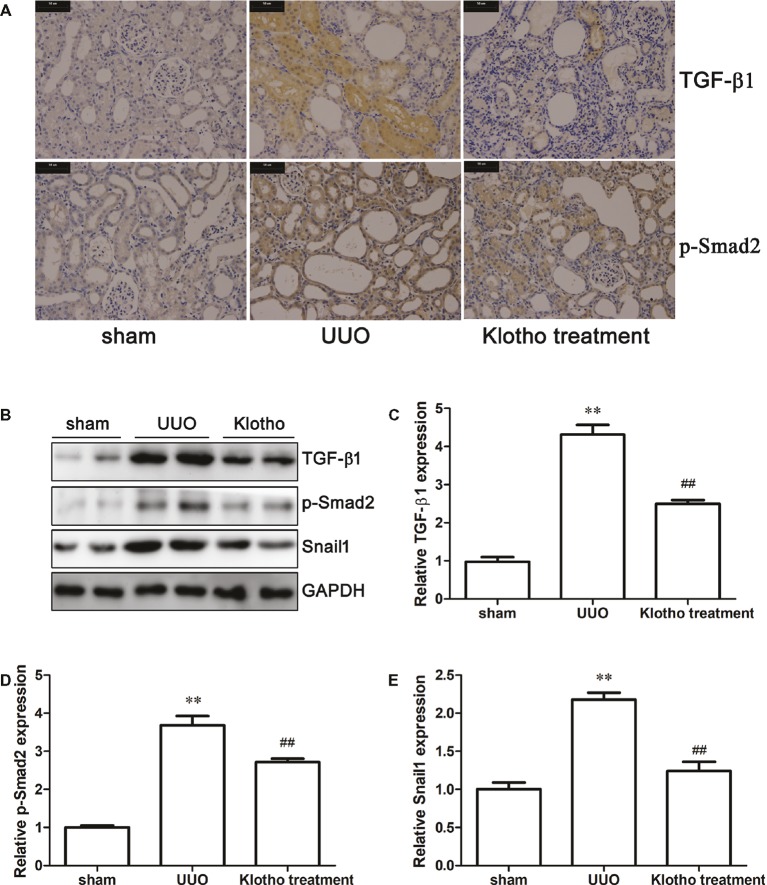
Klotho administration attenuates UUO-triggered EndoMT *via* regulating TGF-β1/Smad2/Snail1 signaling pathway. **(A)** Immunohistochemical staining showing the expression of TGF-β1 and p-Smad2 (S255) in experimental groups. **(B)** Representative images of TGF-β1, p-Smad2 (S255), and Snail1 were analyzed by western blotting. Quantification of TGF-β1, p-Smad2 (S255), and Snail1 is shown in **(C)**–**(E)**, respectively. **p ≤ 0.01 versus sham group; ^##^ *p* ≤ 0.01 versus UUO group.

Immunohistochemistry revealed that the extent of TGF-β1 and p-Smad2 (S255) expression in UUO mice was much stronger than in sham-operated mice (Figure 3A). Western blotting data further confirmed our findings that UUO considerably increased the expression of TGF-β1, p-Smad2 (S255), and Snail1 (Figures 3B–E). These results demonstrated that UUO activated TGF-β1/Smad2/Snail1 signaling, which was at least partially responsible for the initiation of EndoMT. Intriguingly, Klotho treatment decreased levels of TGF-β1, p-Smad2 (S255), and Snail1 to different degrees (Figures 3A–E). These findings indicated that Klotho effectively repressed activation of the TGF-β1/Smad2/Snail1 signaling pathway, which was implicated in the EndoMT process.

## Discussion

We demonstrated exogenous Klotho supplement attenuated renal fibrosis by regulating TGF-β1-induced EndoMT. Our current findings represent a novel mechanism underlying the protective effect of Klotho against renal fibrosis *in vivo*.

Renal fibrosis is characterized by the accumulation of activated myofibroblasts in the kidney. EndoMT is proposed to be an important origin of myofibroblasts and contributes to the initiation and development of renal fibrosis (Cruz-Solbes and Youker, 2017; Sanchez-Duffhues et al., 2018). During the EndoMT process, endothelial cells lose endothelial phenotypes, such as expression of VE-cad or platelet endothelial cell adhesion molecule, and acquire myofibroblast features, such as α-SMA or vimentin. These transformed cells become motile and migrate into surrounding tissues, whereby they contribute to organ fibrosis. In the present study, we observed that UUO resulted in severe renal fibrosis, edema, tubular ectasia, and inflammatory cell infiltration. Moreover, it caused accumulation of α-SMA-expressing cells and a loss of VE-cad-expressing cells in fibrotic kidney. These results support the notion that EndoMT was triggered and implicated in the development of renal fibrosis in obstructed kidney.

EndoMT could be regulated in the context of pathological conditions, such as oxidative stress, inflammatory factors and growth factors including TGF-β1, interleukin 1β, tumor necrosis factor α and NF-κB transcription factor through various signaling pathways (Perez et al., 2017). Use of a TGF-β1-neutralizing antibody or Smad3 knockdown led to EndoMT inhibition (Li et al., 2010; Cooley et al., 2014), suggesting that TGF-β1 and its downstream molecules serve as major regulators of EndoMT (Perez et al., 2017). TGF-β1 binds to its receptor, which results in the phosphorylation of Smads (Smad1, 2, 3, 5, and 8). P-Smads translocate to the nucleus, whereby they regulate the transcription of genes related to fibrosis. Activation of TGF-β1/Smad signaling potently ignites the EndoMT process by upregulating transcription factors Snail1, Snail2, Slug, or Twist by Smad-dependent and Smad-independent pathways (Kokudo et al., 2008; Pardali et al., 2017; Sanchez-Duffhues et al., 2018). These regulatory factors cause the transformation of endothelial cells into mesenchymal cells and lead to the overproduction of myofibroblasts by loss of endothelial markers and acquisition of mesenchymal markers (Kokudo et al., 2008; Piera-Velazquez and Jimenez, 2012). In accordance with this finding, we also found that UUO-induced EndoMT was accompanied by increased expression of TGF-β1, p-Smad2, and Snail1. Such changes in TGF-β1 and its downstream molecules in response to UUO implicate enhanced TGF-β1 signaling in the pathogenesis of EndoMT in fibrotic kidney. Therefore, we can implicate that these signaling pathways may be the target for renoprotection.

Klotho exerts kidney-protective effects and its beneficial actions are multifactorial, including suppression of oxidative stress, inflammation, apoptosis, and organ fibrosis (Dalton et al., 2017; Kuro, 2017). As Klotho is produced primarily in kidney (Hu et al., 2016), it seems plausible that Klotho deficiency is a source of organ disease (Shimamura et al., 2012; Rotondi et al., 2015). Our recent study demonstrated downregulated Klotho expression in CKD, while decreased sKlotho expression increased the risk of creatinine doubling or renal replacement therapy in advanced CKD patients (Liu et al., 2018). In the present study, we observed that UUO caused downregulation of Klotho expression. Furthermore, reduced Klotho levels strengthened TGF-β1 signaling, as indicated by increased TGF-β1, p-Smad2 (S255), and Snail1, thereby implicating Klotho in renal fibrosis at least in part by regulation of TGF-β1 signaling. Actually, there is convincing evidence that Klotho can bind to the TGF-β1 type-II receptor, thereby contributing to the inhibition of TGF-β1 signaling (Doi et al., 2011; Sugiura et al., 2012) and our results were in accordance with this. Additionally, enhanced TGF-β1 signaling was followed by upregulation of α-SMA and downregulation of VE-cad, indicating that EndoMT was activated and involved in fibrotic kidney. Klotho administration dramatically inhibited the renal EndoMT process by downregulating α-SMA and upregulating VE-cad. Most importantly, Klotho treatment suppressed TGF-β1 signaling by regulating TGF-β1 and its downstream effectors. Therefore, our results demonstrated that Klotho inhibits the EndoMT process, which may be beneficial to delay renal fibrosis and this anti-fibrotic effect was possibly through the blockade of TGF-β1 signaling, thus indicating that Klotho protects against renal fibrosis.

In summary, our findings show that attenuation of renal fibrosis by exogenous Klotho might be associated with depressing TGF-β1-mediated renal EndoMT, thus it may be implicated that Klotho could be developed as a target for renal fibrosis in the future. Further studies are required to validate the relationship of Klotho and EndoMT by genetic and pharmacological manipulation.

## Ethics Statement

This study was carried out in accordance with the recommendations of the Institutional Animal Care and Use Committee, Kunshan First People’s Hospital Affiliated to Jiangsu University. The protocol was approved by the ethics committee of Kunshan First People’s Hospital Affiliated to Jiangsu University.

## Author Contributions

SL and QL conceived the study and wrote the manuscript. LY and SL performed biochemical analysis and animal experiments. SL, LY, and AH contributed to histopathological examination, immunofluorescence, immunohistochemistry, and western blotting analysis. All authors edited the manuscript, approved the data and the final version for submission.

### Conflict of Interest Statement

The authors declare that the research was conducted in the absence of any commercial or financial relationships that could be construed as a potential conflict of interest.
